# Digitalization and resilience enhancement: How digital infrastructure construction affects urban resilience

**DOI:** 10.1371/journal.pone.0339790

**Published:** 2025-12-30

**Authors:** Bo Lu, Ruyi Shi, Yibao Wang

**Affiliations:** School of Public Policy and Management (School of Emergency Management), China University of Mining and Technology, Xuzhou, China; Rikkyo University: Rikkyo Daigaku, JAPAN

## Abstract

Enhancing urban resilience is critical for mitigating disaster risks. This study investigates the relationship between digital infrastructure construction (DIC) and urban resilience using panel data from 283 cities in China (2011–2022). Employing constructed indices for DIC and multi-dimensional urban resilience, we empirically analyze the mechanisms and spatial spillover effects of DIC. The study finds: (1) DIC significantly enhances urban resilience, with this positive effect mediated through improvements in critical public services, technological innovation, and resource allocation efficiency. (2) The resilience-enhancing impact of DIC exhibits significant heterogeneity, varying substantially across regions and industrial structures. (3) DIC generates positive spatial spillovers, boosting urban resilience not only locally but also in neighboring regions. These findings provide robust empirical evidence for leveraging DIC as a strategic tool to strengthen urban resilience against disasters.

## Introduction

With the accelerated advancement of China’s new urbanization process, cities have become a complex arena where various disasters and risks are superimposed and interconnected [1]. For one thing, due to the relatively lagging capacity of urban infrastructure to bear and respond to risks, the slow progress of urban governance transformation makes it difficult to effectively address the increasingly complex and dynamic urban risks [2]. For another, under the influence of traditional urban governance models, path dependency characterized by solving problems only as they arise struggles to sustain itself in the face of new challenges [3]. Enhancing the resilience of cities to withstand risk shocks has become an urgent need. Reflecting this imperative, urban resilience development has ascended to paramount priority within recent national policy agendas. Consecutive national policy blueprints, notably the 14th Five-Year Plan and the 20th Party Congress report, explicitly mandate the advancement of resilient cities. With resilience emerging as a pivotal benchmark for effective urban management and safety, it is fundamentally reshaping China’s forthcoming urban development paradigms. Urban resilience signifies an evolution in risk governance, shifting focus from managing known ‘risks’ towards navigating ‘uncertain risks,’ metaphorically embodying a governance logic transition from rigidity towards adaptability [4]. Thus, investigating pathways to augment urban resilience stands as a critical imperative in contemporary urban studies and policy.

The ongoing digital transformation has spawned an innovative paradigm for urban evolution. In the face of inherent vulnerabilities and the complex coupling of urban systems with risks, urban digitalization and smart growth have emerged as pivotal pathways to enhance urban resilience [5]. The digitization and smart development of both the urban physical and social systems can enhance the city’s intelligence, redundancy, and interdependence when facing external shocks [6]. Significantly, in November 2024, the Chinese government promulgated the Guiding Opinions on Advancing New Urban Infrastructure Development for Resilient Cities. This policy mandates the accelerated deployment of digitally integrated, interconnected, and intelligent urban infrastructure systems, aiming to establish highly adaptive and rapidly recoverable resilient cities. DIC is the solid foundation for the digital and smart transformation of cities and serves as the foundational support for urban resilience building and smart governance. In contrast to traditional infrastructure, digital infrastructure is characterized by connectivity, integration, and innovation, based on next-generation information technologies such as 5G, AI, and cloud computing, enabling the iterative upgrading of traditional infrastructure into digitalized, networked, and intelligent systems [7]. Integrating digital infrastructure deeply with urban risk governance scenarios and leveraging digital technologies to quantify and constrain various risks can effectively enhance the responsiveness and adaptability of urban systems, ensuring their functionality, connectivity, and stability. How does DIC empower urban resilience, and are there differences in its empowering effects? What is the mechanism of its effect? Systematically answering these questions provides important insights for improving urban resilience and guiding sustainable urban development.

## Literature review

The term “resilience” was originally a physical concept, referring to the ability of an object or system to return to its original form after deformation caused by external forces. The 1970s witnessed the introduction of the concept of resilience into the field of ecology by Holling, who used it to denote an ecosystem’s capacity to sustain functionality following a disturbance [8]. This marked the genesis of contemporary resilience theory. This concept was later applied to the field of urban studies, shaping the notion of urban resilience. The conceptualisation of this framework underwent a progression from engineering resilience, through ecological resilience, to evolutionary resilience [9]. Early studies primarily focused on the engineering resilience of cities in response to individual natural disasters, emphasizing the physical disaster resistance and recovery capacity of urban infrastructure and other physical components. Today, the concept of urban resilience has significantly expanded, evolving into a multidimensional and systematic framework. Urban resilience refers to an urban system’s ability to maintain its core functions, adapt to changes, and recover rapidly when faced with internal and external disturbances and shocks [10]. At its core, urban resilience aims to enhance the stability and sustainability of urban systems, ensuring that cities can achieve sustainable development amid uncertain environments [11]. Current research on urban resilience primarily focuses on theoretical frameworks, assessment methodologies, and influencing factors. In the study of theoretical frameworks, varying interpretations of resilience across disciplines have led to diverse perspectives and a range of conceptual models. For example, Cutter et al. proposed the Disaster Resilience of Place (DROP) framework, which centers on disaster risk governance and emphasizes infrastructure redundancy, resource availability, and reflective capacity in response to disasters [12]. Some scholars, drawing on complex adaptive systems theory (CAS), have developed frameworks such as HES and RATA that focus on the dynamic responses of urban systems to risks and disturbances [13,14]. Research on urban resilience evaluation has long been a central focus in the field. Its primary objective is to quantify the resilience levels of cities across various dimensions, thereby providing a foundation for enhancing urban resilience. Commonly employed evaluation models and methods include the comprehensive index evaluation method, model simulation, scenario analysis, and resilience network analysis. Furthermore, numerous scholars have attempted to develop urban resilience evaluation index systems; however, a unified framework has yet to be established. Typically, academia constructs these index systems from two perspectives: first, by treating cities as complex coupled systems and developing comprehensive index systems based on urban subsystems such as the economy, society, and ecology [15]; and second, by building index systems from the dynamic perspective of resistance, recovery, and adaptation capabilities or risk response processes, grounded in urban resilience theory [16]. Urban resilience is influenced by a multitude of factors, which scholars generally categorize into human and environmental dimensions. On the human side, key factors include policy and institutional adjustments, economic restructuring, social development levels, and technological innovation [17,18]. From an environmental perspective, studies often examine ecological conditions, natural disasters, public health events, and climate adaptation, exploring how these elements shape resilience and potential pathways for its enhancement [19,20]. Furthermore, with the increasing application of spatial econometric methods in urban studies in recent years, scholars have begun to focus on the spatial effects of urban resilience. Research has revealed that urban resilience is not only influenced by local factors but also exhibits significant spatial dependence and spillover effects. Economic linkages, knowledge spillovers, resource flows, and the cross-regional propagation of disaster risks between neighboring cities may all impact a city’s resilience level [21–23].

As a fundamental part of modern infrastructure systems, digital infrastructure acts as the primary enabler for realizing urban digital and intelligent transformation. Conceptually, digital infrastructure refers to an infrastructure system driven by data innovation, underpinned by communication networks, and centered on data and computing facilities [24]. Distinct from traditional infrastructure predominated by physical entities such as highways and bridges, DIC serves as the technical carrier supporting the operation of digital cities, and is characterized by technology-driven, ubiquitous connectivity, high-speed intelligence, and integrated interconnection. Current academic discourse on digital infrastructure construction primarily focuses on its economic, social, environmental, and innovative impacts. From an economic perspective, DIC stimulates growth, facilitates the flow of production factors, enhances productivity, and optimizes industrial structures [25,26]. For social effects, DIC reduces barriers to information access and strengthens the social inclusion of marginalized groups, thereby playing a significant role in bridging the digital divide, narrowing income disparities, and promoting social equity [27,28]. Regarding environmental effects, DIC contributes to reduced pollution emissions by fostering green innovation and optimizing industrial structures. It also significantly enhances intensive land use in urban areas through smart planning, refined management, and the expansion of virtual spaces, thereby mitigating the encroachment of urban expansion on ecological areas [29,30]. In terms of innovation impact, DIC promotes the transformation of innovation paradigms, lowers the costs associated with innovation, and improves both the efficiency and quality of innovative activities by encouraging digital investment and the adoption of digital technologies [31]. Existing studies utilize various evaluation methods for DIC, which can be broadly classified into two categories: First, evaluations rely on the effects of policy shocks. Scholars use national strategies such as “Smart City” and “Broadband China” as quasi-natural experimental settings [32]. By applying the difference-in-differences (DID) model, they empirically analyze the impact of these policies on the development of digital infrastructure. Second, research is conducted based on indicator quantification [24]. This approach measures the development level of digital infrastructure using single indicators or combinations of multiple indicators. Subsequently, analytical tools such as the fixed effects model (FEM) and the spatial Durbin model (SDM) are employed to explore the effectiveness of DIC.

Although research on digital infrastructure and urban resilience has matured as independent fields, systematic analyses of their interrelationships remain relatively limited. For example, Cheng et al., leveraging the “Broadband China” pilot policy, found that digital infrastructure enhances urban economic resilience through industrial structure optimization and the enabling effects of digital finance [33]. Similarly, Jiang et al. used a DID model to demonstrate that telecommunications infrastructure significantly strengthens urban economic resilience, with effects growing more pronounced over time [34]. While such studies confirm a positive correlation between digital infrastructure and economic resilience, they offer limited insight into how digital infrastructure holistically enables resilience across the entire urban system. A small number of scholars have also attempted to explore the relationship between the two from the spatiotemporal dimension or through macro-theoretical descriptions. For instance, Zhang et al. employed a coupling coordination degree model to evaluate the coordination between new infrastructure and regional resilience in China, identifying an “east high, west low” spatial pattern [35]. However, their study remains descriptive and does not explain the causes of this disparity. Likewise, Sajjad et al. qualitatively suggested that digital technologies can enhance disaster resilience in high-density urban areas by improving monitoring accuracy, yet their proposition lacks empirical validation [36].

To summarize, existing studies have laid a foundation for analyzing the relationship between DIC and urban resilience. Nevertheless, significant research gaps remain, which can be specified as follows: Firstly, existing literature exhibits insufficient attention to DIC—a novel variable. Most studies focus on traditional physical infrastructure or policy, and are yet to systematically reveal the mechanism through which DIC enhances urban resilience across multiple dimensions. Secondly, most existing studies analyze the impact of DIC on a single domain in isolation, failing to examine it within the framework of improving the overall resilience of urban systems. Among the small number of studies that involve DIC and urban resilience, the focus is either limited to the single dimension of economic resilience or remains confined to descriptions of spatiotemporal relationships. These studies have not moved beyond phenomenon-based analysis, resulting in insufficient depth in exploring the intrinsic functional pathways between DIC and urban resilience. Thirdly, from the perspective of research methods and approaches, most existing studies rely on single-case analyses, provincial-level macroeconomic data, or employ policies such as “Smart City” and “Broadband China” as quasi-natural experiments, typically using DID model to assess the impact of digital infrastructure development. However, there is a relative paucity of research that constructs a comprehensive indicator system at the urban scale to measure the level of DIC and examine its effects on urban resilience. This hinders the accurate elucidation of the potential mechanisms and regional heterogeneity through which DIC impacts urban resilience at the city level. Therefore, it is essential to explore, both theoretically and empirically, the influence and inherent mechanisms of DIC with respect to urban resilience. The potential contributions of this study reside in: exploratorily constructing a theoretical analytical framework for the empowerment of urban resilience through digital infrastructure development, and systematically uncovering the multi-layered influence pathways and dynamic transmission mechanisms by which DIC impacts urban resilience—via the integration of econometric model verification and empirical evidence. The aim is to enrich and improve research on resilient city construction, providing theoretical support and practical considerations for future urban resilience development.

## Theoretical analysis and research hypotheses

### Direct effects of DIC on empowering urban resilience

Urban resilience involves a coupled system across multiple aspects, including the economy, society, ecology, and infrastructure [26]. Digitalization provides new technological conditions and development models for building resilient cities, which can enhance the resilience capacity of various urban subsystems [37]. Therefore, this paper analyzes the direct effects of DIC on empowering urban resilience from four dimensions: economic resilience, social resilience, ecological resilience, and infrastructure resilience.

Firstly, from the dimension of economic resilience, digital infrastructure, as a core driver of the digital economy, is capable of activating urban economic vitality and thus strengthening urban economic resilience [34]. The DIC fosters the thorough implementation of digital technologies, propelling the informatization, digitization, and intelligent transformation of traditional industries. This process elevates both the standard and effectiveness of economic advancement, concurrently strengthening the long-term stability and the ability to adapt of urban economic setups. The DIC has given rise to new digital business models, enriching the urban industrial structure. The characteristics of emerging industries, such as high value-added, innovation, and growth potential, can bring new momentum and opportunities to urban economies, strengthening their resilience to risks [38]. By leveraging the network effects inherent in digital infrastructure, it is possible to expedite industrial reorganization and supply chain collaboration. This process facilitates the profound integration of digital and real economies, thus further strengthening the resilience of urban economies.

Secondly, regarding social resilience, the construction and application of digital infrastructure provides more efficient and diverse governance tools and approaches for governments and social organizations [39]. On the one hand, through technologies such as the IoT, big data, and cloud computing, it enables holistic perception and precise analysis of urban operations, strengthens urban weaknesses, and enhances the scientific nature, foresight, and responsiveness of urban management [18]. On the other hand, the DIC is a key driving force for innovating urban governance models [40]. By empowering the construction of smart city platforms through ‘digital+’, it facilitates information exchange in social operations and solving the problem of data silos, improving social governance efficiency and transparency, and enhancing the level of refined urban governance and urban social resilience overall.

Additionally, at the level of ecological resilience, digital infrastructure, by promoting green development and sustainability, enhances the diversity and stability of urban ecosystems [41]. Through the digitalization of industries, it drives the transformation and upgrading of traditional industries, continuously reshaping a low-carbon industrial structure, promoting resource conservation and environmental protection, and enhancing ecological resilience [42]. Through intelligent monitoring and data analysis, it enables precise protection and management of the ecological environment, coordinating the relationship between economic and social development and ecological conservation, providing strong support for enhancing urban ecological resilience [43].

Finally, in terms of infrastructure resilience, the DIC, through the renovation of traditional infrastructure, builds intelligent and networked infrastructure systems, enhancing the reliability and redundancy of urban infrastructure, improving its operational efficiency and management level [44]. The integration of digital technologies promotes information sharing and collaborative integration between different infrastructures, by reasonably adjusting the redundancy and availability of similar functional components, which not only strengthens the overall effectiveness of infrastructure but also further enhances its resilience and adaptability in the face of extreme weather, natural disasters, and other external challenges [45].

Therefore, we propose the following hypotheses:

H1: DIC has a positive impact on urban resilience.

### Indirect effects of DIC in empowering urban resilience

Digital infrastructure is a new type of infrastructure characterized by networking, digitization, and intelligence, which still retains the public good attributes of traditional infrastructure and serves as a supportive facility for providing public goods and services [46]. Based on the theory of public goods, digital infrastructure can enrich the supply models and types of public goods and services through digital networks and shared platforms, facilitating enhancements in the quality and efficiency of public service provision [47]. The theory of endogenous growth posits that technological advancement and its positive externalities are key factors in achieving regional endogenous growth [48]. Digital infrastructure, as the material carrier of digital technologies, enables the application and advancement of technology to trigger the ‘technological effect’ of knowledge spillover and technological diffusion, as well as the ‘structural effect’ of adjusting the factor resource structure [49,50]. Therefore, this paper explores the indirect mechanisms through three paths: basic public services, technological innovation, and resource allocation, to examine the indirect mechanisms through which digital infrastructure empowers urban resilience.

#### DIC enhances urban resilience through optimizing basic public services.

DIC not only transforms the supply model of basic public services by enabling the cross-regional flow of basic public service resources and enhancing the accessibility of public services, but also strengthens information interconnection, alleviates information asymmetry, and improves the precision of matching supply and demand for public services. Within this context, it provides resilient support for cities in the face of disturbances. First, DIC facilitates the digital transformation of basic public services, allowing such services to transcend geographical boundaries in the form of data, expand service coverage, and enhance the accessibility of basic public services [51]. By converting the physical carriers of public services into digital ones, DIC eliminates hardware gaps between urban and rural areas as well as across regions, driving a shift in public services from partial coverage to universal access across the entire region, and from over-reliance on offline channels to round-the-clock service integrating online-offline collaboration. This cross-regional and round-the-clock supply of public services directly enhances cities’ capacity to respond to crises, maintain operations, and achieve rapid recovery [52]. Second, digital technologies reshape the supply model of basic public services. Through the application of large-scale platforms and big data, a service ecosystem characterized by multi-stakeholder participation, mutual trust, and interconnection is established [53]. This ecosystem alleviates information asymmetry and further improves the efficiency of public services. The development of digital platforms facilitates the accurate identification of public service demands and preferences, promotes seamless connection between the supply and demand of basic public services, mitigates imbalances in the supply structure of basic public services, and enhances the efficiency of basic public service supply [54]. Third, digital infrastructure enhances the dynamic adaptability of basic public services. Public service systems supported by key technological infrastructures such as 5G networks and industrial internet exhibit high flexibility and reconfigurability. They can achieve real-time perception of and rapid response to demands, dynamically adjust the scale of public services, and realize precision supply and optimized resource allocation. When cities are impacted by emergent incidents, digital public service systems can quickly switch to emergency mode to maintain uninterrupted basic urban functions. Moreover, through intelligent response mechanisms, these systems effectively mitigate social fluctuations, supporting cities in achieving rapid recovery and functional reconstruction.

#### DIC empowers urban resilience through promoting technological innovation.

As the “hardware foundation” for urban digital development, digital infrastructure, which encompasses core components such as 5G base stations, data centers, industrial internet, and computing power networks, has emerged as a critical supporting force for driving technological innovation and the development of urban resilience. On the one hand, compared with the single-functional nature of traditional infrastructure, DIC provides fundamental conditions and extensive application scenarios for technological innovation by constructing high-speed interconnected data circulation networks and computing power support systems [55]. This, in turn, systematically promotes technological iteration and integrated innovation. On the other hand, driven by the dual goals of smart governance and sustainable development, achievements in technological innovation (e.g., intelligent technologies and smart devices) are transformed into key instruments for urban resilience construction through integration with scenarios such as urban management, facility operation, and emergency response [18]. Existing studies have shown that technological innovation is the core driver of enhancing urban resilience. For one thing, the innovative application of digital technologies significantly improves cities’ risk perception and emergency response capabilities [56]. By leveraging new-generation information technologies such as 5G, AI, and cloud computing, cities can achieve real-time dynamic monitoring of key systems (e.g., environment, transportation, and energy), effectively identify potential risks, and implement early warnings—thereby reducing urban vulnerability. For another, technological innovation strengthens the adaptability and resilience of urban systems: technologies such as drone inspections and robotic emergency rescue enhance on-site disaster response capabilities, while telemedicine and online education ensure the continuity of public services during special periods [52]. Thus, in the process of promoting technological innovation, the DIC can indirectly enhance the overall resilience level of cities by strengthening risk prevention and control, optimizing service provision, and improving emergency response capabilities.

#### DIC enhances urban resilience by optimizing resource allocation efficiency.

In the past, inefficient or mismatched resource allocation during urban emergencies often led to delayed emergency responses and slowed recovery of urban functions [57]. DIC addresses these issues by alleviating information asymmetry, breaking down barriers to resource flow, and optimizing the core logic of resource allocation. This process reduces idle and misallocated resources, thereby enhancing the flexibility and coordination of urban systems. First, DIC enhances the mobility and permeability of information and knowledge, mitigating information asymmetry. Through the establishment of comprehensive and intelligent digital platforms supported by technologies such as the Internet of Things, big data, and artificial intelligence, DIC enables real-time monitoring of urban elements—including human resources, materials, and energy—as well as accurate identification of demand [58]. This capability allows governments and market entities to avoid redundant investments or local shortages caused by delayed or incomplete information, thereby improving the precision and efficiency of resource allocation. Second, DIC breaks down administrative and physical barriers that traditionally hinder resource mobility, promoting efficient circulation and cross-domain integration of production factors [59]. By establishing integrated, cross-departmental, and cross-regional resource coordination platforms, DIC overcomes institutional obstacles caused by fragmented management and bureaucratic segmentation. Resources can be dynamically allocated among different entities and regions based on actual needs, significantly reducing sunk resources and mobility constraints caused by institutional mechanisms and geographical limitations [60]. This enhances the city’s capacity for resource reorganization and rapid response in emergency situations. Consequently, DIC not only reduces frictional costs and efficiency losses in urban system operations but also strengthens synergies among multiple subsystems when confronting emergencies. This helps maintain functional stability and facilitate rapid recovery in uncertain environments. Therefore, through improving resource allocation efficiency, mitigating information asymmetry, and enhancing coordination and emergency response capabilities, digital infrastructure construction indirectly contributes to the overall resilience of cities.

Based on this evidence, we propose the following hypotheses:

H2: DIC empowers urban resilience by optimizing basic public services, promoting technological innovation, and improving resource allocation efficiency.

### Spatial effects of DIC on empowering urban resilience

Digital infrastructure not only has strong penetration and integration characteristics but also possesses externalities, network characteristics, and shared features [61,62]. DIC plays a crucial role in breaking down spatial and temporal barriers between cities, accelerating factor spillover, and promoting inter-regional exchange and cooperation [63]. On the one hand, compared to traditional infrastructure, digital infrastructure strengthens the speed, breadth, and depth of technological diffusion. As digital infrastructure improves, the ‘information bridge’ between regions and cities becomes increasingly smooth, and the dissemination and application of new technologies across different industries not only enhances the resilience capacity of the region but also empowers surrounding cities through the effect of technological diffusion, further improving their resilience. On the other hand, DIC strongly promotes regional collaboration and resource sharing, strengthening cooperation, sharing, and synergy between cities, forming a pattern of complementary advantages and coordinated development. Through deepening inter-regional cooperation and coordination, it not only enhances overall regional competitiveness but also effectively addresses complex cross-regional issues, thereby strengthening urban resilience. Based on the above, the following hypothesis is proposed:

H3: The empowerment effect of DIC on urban resilience has spatial spillover effects.

**Fig 1:**
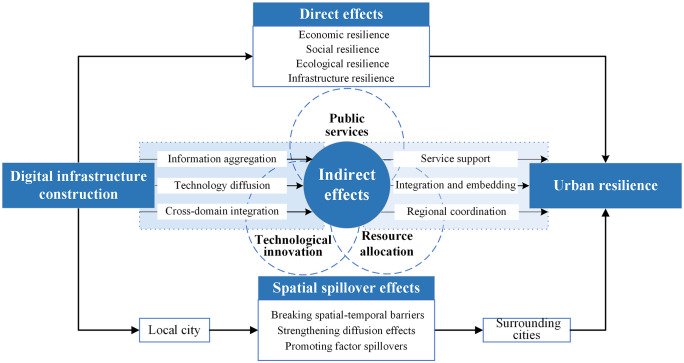
The mechanism of digital infrastructure construction in empowering urban resilience.

Based on the above, this study explores the mechanisms through which DIC influences urban resilience across three key dimensions: direct impacts, indirect pathways, and spatial effects. The corresponding theoretical framework is depicted in Fig 1.

## Empirical design

### Data

This study utilizes a panel of 283 Chinese cities spanning the period 2011–2022. Primary data were sourced from the publicly released *China Statistical Yearbook, China City Statistical Yearbook*, and *China Urban Construction Statistical Yearbook*, supplemented by provincial/municipal statistical yearbooks and development bulletins. Missing values were addressed through linear interpolation and ARIMA imputation techniques.

### Model

#### Benchmark regression model.

Following the Hausman test, a panel model with two-way fixed effects is developed to examine whether digital infrastructure development positively influences the enhancement of urban resilience, thereby verifying research hypothesis H1 proposed in this study.


$${UR}_{i,t}=a_{\text{0}}+a_{\text{1}}{DIC}_{i,t}+a_{c}Z_{i,t}+\mu_{i}+\delta_{t}+\varepsilon_{i,t}$$ (1)

In Equation (1), *UR*_*i,t*_ represents the urban resilience level of city i in year t; *DIC*_*i,t*_ represents the level of digital infrastructure construction in city i in year t; *Z*_*i,t*_ represents a set of control variables involved in this study’s empirical analysis; *μ*_*i*_ and *δ*_*t*_ represent the individual fixed effects and time fixed effects, respectively, while *ε*_*i,t*_ is the random disturbance term.

#### Mediation effect model.

To investigate the potential mechanisms through which DIC enhances urban resilience, and to test research hypothesis H2, this study draws on the work of Li et al. and further extends model (1) to construct models (2) and (3). A mediation effect analysis is employed for empirical verification. Specifically, if the regression coefficients β1, γ1, and γ2 demonstrate significant positivity, it implies that the mediator variable exerts a partial mediating influence on the relationship between DIC and urban resilience.


$$M_{i,t}=\beta_{\text{0}}+\beta_{\text{1}}{DIC}_{i,t}+\beta_{c}Z_{i,t}+\mu_{i}+\delta_{t}+\varepsilon_{i,t}$$ (2)

$${UR}_{i,t}=\gamma_{\text{0}}+\gamma_{\text{1}}{DIC}_{i,t}+\gamma_{\text{2}}M_{i,t}+\gamma_{c}Z_{i,t}+\mu_{i}+\delta_{t}+\varepsilon_{i,t}$$ (3)

Where *M*_*i,t*_ is the mediator variable, and the meanings of the other variables are consistent with those in Equation (1).

#### Spatial econometric model: Spatial durbin model.

This study uses the Spatial Durbin Model (SDM) to identify the spatial effects present, as shown in model (4).


$${UR}_{it}=a_{\text{0}}+\rho_{\text{1}}W\times {UR}_{i,t}+a_{\text{1}}{DIC}_{i,t}+a_{c}Z_{i,t}+\theta_{\text{1}}W\times {DIC}_{i,t}+\theta_{\text{1}}W\times Z_{i,t}+\mu_{i}+\delta_{t}+\varepsilon_{i,t}$$ (4)

In Equation (4), *ρ*_*1*_ denotes the estimated parameter for the spatial lag coefficient of the dependent variable. W represents the spatial weights matrix, and all other variables retain their definitions from Equation (1).

### Variable selection and measurement

#### Dependent variable: Urban resilience.

Urban resilience is defined as a city’s comprehensive capacity to effectively withstand shocks, absorb losses, and rapidly recover when facing various pressures, risks, and threats, ultimately maintaining the dynamic equilibrium and sustainable development of the urban system. This includes resilience in the economic, social, ecological, and infrastructure aspects of the city. Therefore, this research integrates the conceptual framework proposed by Jiang et al. [17] and Zhou et al. [64], conceptualizing urban resilience through its four constitutive pillars: ecological, social, economic, and infrastructural resilience. A corresponding indicator framework was developed based on this dimensional structure (detailed in Table 1). To address the influence of indicator dimensions and their inherent properties, both positive and negative indicators underwent normalization, and the entropy weight technique was utilized to assign weights to these indicators.

**Table 1:** Urban resilience evaluation indicator system.

Variable	Dimension	Specific indicators	Indicator attribute
Urban resilience (UR)	Economic resilience (UER)	Per capita regional GDP (CNY)	**+**
		Average wage of employees (CNY)	**+**
		Loan-to-GDP ratio of financial institutions at year-end (%)	**+**
		Per capita fixed asset investment (CNY)	**+**
		Science and technology expenditure as a percentage of general public budget (%)	**+**
		Per capita retail sales of consumer goods (CNY)	**+**
	Social resilience (USR)	Number of university students per 10,000 population	**+**
		Natural population growth rate (‰)	**+**
		Per capita savings deposits of urban and rural residents (CNY)	**+**
		Social security expenditure as a share of total expenditure (%)	**+**
		Tertiary sector employment share (%)	**+**
		Public administration and social organizations employment share (%)	**+**
	Ecological resilience (UELR)	Municipal solid waste treatment rate (%)	**+**
		Centralized sewage treatment rate (%)	**+**
		Urban built-up area green coverage rate (%)	**+**
		Per capita park green space area (m²)	**+**
		Industrial wastewater discharge (10⁴tons)	**–**
		Annual average concentration of inhalable fine particulate matter (PM_2.5_, μg/m³)	**–**
	Infrastructure resilience (UIR)	Number of hospitals and primary care clinics	**+**
		Public transport vehicles per 10,000 population	**+**
		Road network density in built-up areas (km/km²)	**+**
		Daily production capacity of urban public water supply (10⁴m³/day)	**+**
		Total length of urban drainage pipelines (km)	**+**
		Completed fixed asset investment in municipal utilities (CNY)	**+**

#### Explanatory variable: Digital infrastructure construction.

Digital infrastructure is a systematic project, and thus a single indicator cannot comprehensively measure the level of DIC. Accounting for data accessibility constraints at the city level and building upon the methodological frameworks of Wang et al. [65] and Hu et al. [66], this study established a digital infrastructure evaluation index system(Table 2). The system comprises six indicators structured into two dimensions: digital infrastructure investment and output. Subsequently, the DIC Index was calculated using the entropy weighting method.

**Table 2:** Digital infrastructure construction evaluation indicator system.

Variable	Dimension	Specific indicators	Indicator attribute
Digital infrastructure construction index (DIC)	Digital infrastructure input	Fiber optic cable density	**+**
		Per capita internet access broadband ports (units)	**+**
		Mobile communication base station density	**+**
		Proportion of workforce in ICT and software industries (%)	**+**
	Digital infrastructure output	Telecommunication service revenue (CNY)	**+**
		Mobile phone penetration rate (%)	**+**
		Internet penetration rate (%)	**+**
		Digital inclusive finance index	**+**

Note: Due to the unavailability of optical cable line length and mobile communication base station data at the prefecture-level city level, provincial-level data for these indicators were apportioned to individual cities based on each city’s share of total telecommunications revenue within its respective province. The detailed calculation is defined as follows: Let ${\text{X}}_{\text{province}}$ represent the total provincial-level data of a target indicator; ${\text{R}}_{\text{city}}$ represent the telecommunications revenue of a specific prefecture-level city within the province; and ${\text{R}}_{\text{total},\text{province}}$ represent the sum of telecommunications revenues of all prefecture-level cities in the province. Then, the apportioned value of the target indicator for the specific prefecture-level city (${\text{X}}_{\text{city}}$) is calculated using the formula: ${\text{X}}_{\text{city}}={\text{X}}_{\text{province}}\times \frac{{\text{R}}_{\text{city}}}{{\text{R}}_{\text{total},\text{province}}}$

#### Mediating variables.

This study selects three indicators—basic public services, technological innovation, and resource allocation—as mediating variables. The public service index is based on the research of Zhang et al. [67], which includes indicators from education, healthcare, and social security, using ratios such as the teacher-student ratio at different education stages, the number of doctors and hospital beds per 10,000 people, and the ratio of people insured in pension, medical, and unemployment insurance to the annual average population, all calculated by weighted averages. Technological innovation is based on the research of Li et al. [68], using indicators such as R&D intensity, the number of patents per capita, and the proportion of R&D personnel calculated by weighted averages using the entropy method. Resource allocation efficiency can be reflected by the degree of market distortion. Resource allocation efficiency(RAE), gauged by market distortion, adopts Liu et al.‘s [69] approach. The market distortion level for each city is evaluated through a production function model, and the distortion index is reversed to calculate the RAE for each region.

#### Control variables.

To precisely assess the influence of DIC on urban resilience, this study references existing related research [28,32,67]. It selects four indicators as control variables: urbanization rate, government intervention, industrial structure upgrading, and population density. Specifically, the urbanization rate is computed as the proportion of the urban population to the year-end resident population; governmental intervention is quantified by the percentage of local fiscal budget outlay in regional GDP; industrial structure advancement is denoted by the ratio of the tertiary industry’s value – added to that of the secondary industry; and population density is ascertained as the ratio of the resident population to the land area of the administrative region. The descriptive statistics for the primary variables in this research are displayed in Table 3.

**Table 3:** Descriptive statistics results.

Variables	Symbol	Observation	Mean	Std. Dev.	Min	Max
Urban resilience	UR	3396	0.0970	0.0700	0.0330	0.580
Digital infrastructure construction	DIC	3396	0.0320	0.0390	0.00300	0.744
Public services	PS	3396	0.144	0.0670	0.0300	0.476
Resource allocation efficiency	RAE	3396	1.314	0.817	0.0120	7.907
Technological innovation	TI	3396	0.0390	0.0480	0.00100	0.554
Urbanization rate	UBR	3396	57.33	14.72	21.40	100
Government Intervention	GI	3396	0.202	0.102	0.0440	0.916
Industrial structure upgrading	ISU	3396	1.081	0.612	0.114	5.650
Population density	PD	3396	438.9	347.1	5	2712

## Results and discussion

### Benchmark regression analysis

The model’s multicollinearity was evaluated using the Variance Inflation Factor (VIF). All explanatory variables had VIF values below 10 (mean = 2.01), indicating no significant multicollinearity issues. Subsequently, a Hausman specification test was conducted, which rejected the random – effects null hypothesis. Consequently, a two – way fixed effects model was employed to examine the impact of digital infrastructure development on urban resilience. The detailed in Table 4, Columns (1) and (2) display regression results without control variables. In these columns, the coefficient estimated for digital infrastructure is statistically significant and positive at the 1% level. Columns (3) through (7) show the regression results of digital infrastructure on various urban resilience subsystems, with control variables added based on column (1) while accounting for fixed effects of cities and years. The results in Table 4 indicate that, with the exception of the coefficient for ecological resilience, which is not significant, the coefficients for digital infrastructure’s impact on overall urban resilience and the other three resilience subsystems remain significantly positive. benchmark regression results are presented in Table 4. The insignificance of ecological resilience could be attributed to the reality that advancements in ecological resilience depend more on policies and measures concerning environmental protection, resource preservation, and sustainable development. In these domains, the function of digital infrastructure might be comparatively restricted. Based on the foregoing findings, it is clear that even when adding extra control variables, the effect coefficient of DIC on urban resilience stays notably positive. This offers initial confirmation for the facilitating role of building digital infrastructure in enhancing urban resilience, so verifying Hypothesis H1.

**Table 4:** Benchmark regression results.

Variable	(1)	(2)	(3)	(4)	(5)	(6)	(7)
	UR	UR	UR	UER	USR	UELR	UIR
DIC	1.213***	0.254***	0.236***	0.041***	0.016***	−0.001	0.180**
	(0.023)	(0.090)	(0.085)	(0.013)	(0.005)	(0.001)	(0.073)
Controls	No	No	Yes	Yes	Yes	Yes	Yes
_cons	0.058***	0.070***	0.053***	0.015***	0.025***	0.007***	0.006
	(0.001)	(0.002)	(0.013)	(0.004)	(0.002)	(0.000)	(0.011)
City fixed effect	No	Yes	Yes	Yes	Yes	Yes	Yes
Year fixed effect	No	Yes	Yes	Yes	Yes	Yes	Yes
N	3396	3396	3396	3396	3396	3396	3396
R^2^	0.441	0.536	0.563	0.725	0.313	0.388	0.191

Note: * p < 0.1, ** p < 0.05, *** p < 0.01, with the values in parentheses representing robust standard errors.

### Robustness and endogeneity tests

The findings from the benchmark regression demonstrate that DIC has a significant positive impact on urban resilience. To guarantee the credibility of these findings, this study conducts robustness tests by adding control variables, removing outliers, and employing winsorization methods. Additionally, instrumental variable methods and lagged explanatory variables are used to address potential endogeneity issues.

#### Robustness test.

(1) Adding control variables. To mitigate potential bias from omitted important explanatory variables leading to inaccurate estimates, this study further controls for urban economic agglomeration and openness to foreign trade, in addition to the variables already controlled for in the benchmark regression model. Urban economic agglomeration is quantified as regional GDP per unit land area within administrative boundaries, while foreign trade openness is expressed as the proportion of total import-export value relative to regional GDP. Both variables are integrated into the model for regression analysis, and the findings are presented in column (1) of Table 5.
(2) Cities with special administrative status (including centrally-administered municipalities and provincial capitals) have unique administrative levels, economic status, and policy environments, which may result in significant data differences compared to other cities, potentially leading to biased or inaccurate results. To mitigate potential estimation bias arising from these institutional disparities, our sample excludes 30 such cities (including four centrally-administered municipalities and 26 provincial capitals). The outcomes are presented in Table 5, column (2).
(3) Winsorization of outliers. To address potential distortion from extreme values, this research implements a 1% winsorization across all variables and performs a subsequent re – regression. The relevant results are exhibited in column (3) of Table 5.

**Table 5:** Robustness test results.

Variables	Adding control variables	Changing the Sample Size	Winsorization of outliers
	(1)	(2)	(3)
DIC	0.220***	0.147*	0.573***
	(0.082)	(0.084)	(0.179)
Controls	Yes	Yes	Yes
_cons	−0.062**	0.050***	0.040
	(0.030)	(0.007)	(0.029)
City fixed effect	Yes	Yes	Yes
Year fixed effect	Yes	Yes	Yes
N	3396	3036	3396
R^2^	0.578	0.693	0.582

Note: * p < 0.1, ** p < 0.05, *** p < 0.01, with the values in parentheses representing robust standard errors.

From Table 5, it is evident that although certain parameter estimates underwent changes during the robustness test, the effect of DIC on urban resilience persisted as significantly positive. This outcome attests to the robustness of the benchmark findings and validates that DIC can effectively contribute to the enhancement of urban resilience.

#### Endogeneity test.

(1) DIC can promote urban resilience; however, the research methodology may overlook endogeneity issues arising from omitted variables, which could affect the accuracy of the final results. Thus, drawing on the study by Wang et al. [70], this research uses the number of fixed-line telephones per 100 people in each city in 1984 as an instrumental variable(IV) for digital infrastructure to mitigate endogeneity effects. The primary reason is that the IV represents the early communication infrastructure level of cities, reflecting the foundational communication network, technological adoption capacity, and economic activity of cities in their early stages, thus meeting the relevance requirement for selecting an instrumental variable. Furthermore, the IV is historical data, with no direct causal relationship to current urban resilience, thus avoiding reverse causality and satisfying the exogeneity requirement for the instrumental variable selection. The regression results are presented in columns (1) and (2) of Table 6. The first-stage regression yielded a statistically significant positive coefficient (at the 1% level) for the first lag of DIC. The second-stage analysis demonstrates that DIC’s positive influence on urban resilience remains statistically robust after incorporating the IV. Further validation via the K-P rk Wald F statistic shows it exceeds the 10% critical value (16.38) from the Stock-Yogo weak identification test, indicating a strong correlation between the IV and the DIC, thus ruling out weak instrument concerns. Additionally, the K-P rk LM test rejects the null hypothesis of insufficient instrument identification, verifying the suitability of the variable IV and the robustness of the estimation results
(2) Lag order of explanatory variables. The planning, construction, and commissioning of digital infrastructure involve time cycles, and its empowering effect on urban resilience needs to be realized through the transmission chain of “infrastructure construction—application penetration—resilience enhancement,” which may involve a lag. Therefore, this study applies one-period and two-period lag treatment to the explanatory variables for regression analysis. The estimation results are exhibited in columns (3) and (4) of Table 6. The findings reveal that the regression coefficient for DIC stays remarkably positive, implying that digital infrastructure can efficiently boost the enhancement of urban resilience, which is in consonance with the benchmark regression findings.

**Table 6:** Endogeneity test results.

Variables	Instrumental variables method		Lagged explanatory variable (t-1)	Lagged explanatory variable (t-2)
	First stage (1)	Second stage (2)	(3)	(4)
IV	0.001***			
	(0.000)			
DIC		0.307***		
		(0.0839)		
L.DIC			0.195***	
			(0.0220)	
L2.DIC				0.245***
				(0.0281)
Controls	Yes	Yes	Yes	Yes
Kleibergen-Paap rk LM statistic		18.845***		
Kleibergen-Paap rk Wald F statistic		26.068 {16.38}		
First-stage F-statistic	26.07			
City fixed effect	Yes	Yes	Yes	Yes
Year fixed effect	Yes	Yes	Yes	Yes
N	2676	2676	3113	2830

Note: * p < 0.1, ** p < 0.05, *** p < 0.01, with the values in parentheses representing robust standard errors; the values within {} in the Kleibergen-Paap rk Wald F statistic represent the critical values at the 10% level from the Stock-Yogo test.

### Heterogeneity analysis

#### Regional heterogeneity analysis.

Regional disparities in economic development, industrial structure, and population scale across different geographical areas, coupled with spatial heterogeneity in digital infrastructure deployment, may lead to differential impacts of DIC on urban resilience. To empirically examine this spatial heterogeneity, we conduct subsample analyses by dividing the aggregate sample into three distinct regional cohorts: eastern, central, and western China. As demonstrated in Columns (1)-(3) of Table 7, DIC in eastern China exhibits a statistically significant positive effect on urban resilience at the 5% level, whereas the coefficients for central and western regions remain positive yet statistically insignificant.

**Table 7:** Heterogeneity analysis results.

Variables	Eastern region	Central region	Western region	Industry-driven cities	Service-driven cities
	(1)	(2)	(3)	(4)	(5)
*DIC*	0.154**	0.412	0.304	0.125	0.192**
	(0.0680)	(0.2535)	(0.2656)	(0.0915)	(0.0819)
_cons	0.112***	0.051***	−0.119*	0.072***	0.053**
	(0.0318)	(0.0100)	(0.0662)	(0.0166)	0.0233
Controls	Yes	Yes	Yes	Yes	Yes
City fixed effect	Yes	Yes	Yes	Yes	Yes
Year fixed effect	Yes	Yes	Yes	Yes	Yes
N	1152	1236	1008	1875	1521
R^2^	0.631	0.580	0.592	0.616	0.522

Note: * p < 0.1, ** p < 0.05, *** p < 0.01, with the values in parentheses representing robust standard errors.

The disparity arises not only from the comprehensive advantages of eastern cities in economic fundamentals, policy support, application scenarios, and technology transfer but also from the diffusion law of digital technology and the regional digital divide. On the one hand, the eastern region boasts a strong economic foundation, significant industrial advantages, an early start in digital transformation, and a mature digital ecosystem, all of which facilitate the digital infrastructure in achieving economies of scale and agglomeration effects. Additionally, as one of the earliest policy pilot areas for the national digital strategy, the eastern region has received greater fiscal subsidies and benefits from institutional innovation. Leveraging a high concentration of high-tech enterprises, universities, and research institutes, its diverse talent pool has accelerated the conversion and implementation of technologies. On the other hand, the “digital divide” has created significant disparities between the eastern and central-western regions in terms of technological application capabilities and demand levels. Due to the time-lag effect of technology diffusion, even if the central-western regions gradually catch up in hardware development, it will take considerably longer to realize the full benefits of digital infrastructure. Currently, DIC in the central-western regions remains focused primarily on hardware coverage, with insufficient integration and adaptability to various scenarios. Additionally, the supporting economic, industrial, and talent conditions have not yet reached the threshold necessary to unlock digital dividends, thereby limiting the infrastructure’s potential to significantly enhance urban resilience.

#### Industrial structure heterogeneity analysis.

In consideration of the potential disparities in the repercussions of DIC on the resilience of cities exhibiting disparate industrial configurations, this study employs a classification system that categorises the sample cities according to their industrial structure characteristics. Specifically, the dataset is partitioned into two categories for comparative analysis, using the tertiary-to-secondary industry output ratio as the criterion. Cities with a ratio exceeding 1 are classified as service-dominated cities, while those with a ratio below 1 are designated as industry-dominated cities. Regression results for these groups (Table 7, Columns 4–5) reveal a stark contrast: DIC exerts a significant positive impact on resilience in service-dominated cities but yields no statistically significant effect in industry-dominated cities.

The reason may lie in the fact that the main industries in service-dominated cities, such as finance, logistics, and information technology services, have a natural compatibility with digital technologies, leading to high levels of digital penetration. DIC can expand service coverage, enhance demand response flexibility, and improve urban governance and service efficiency, thereby increasing the city’s ability to cope with external shocks and recover. Industry-dominated cities primarily rely on traditional manufacturing, with an over-reliance on industry in their development models, a relatively homogeneous industrial structure, and a lack of diversified industrial support. This over-reliance, in turn, leads to high dependence of their economic and social structures on industry, leaving these cities deficient in risk resistance across economic, social, and environmental dimensions. Once external shocks occur, the entire urban system is prone to cascading crises. On the one hand, industry-dominated cities often exhibit high dependence on resources, markets, and national policies, resulting in inherent deficiencies in risk resistance. DIC cannot fully replace such rigid constraints, nor can it effectively mitigate the impacts of shocks. For instance, in cases of industrial shutdowns caused by factors like the COVID-19 pandemic, supply chain disruptions, or raw material shortages, DIC has a limited direct effect on alleviating such risks. On the other hand, the long-standing model of relying on industrial growth has fostered a rigid path dependence and governance paradigm in these cities. Resource and policy inputs tend to focus on industrial production links, leading to insufficient application of inclusive DIC for overall urban governance. Lagging digital integration and transformation in areas such as urban emergency management and public services further prevent DIC from fully exerting its role in enhancing comprehensive urban resilience.

### Mechanism analysis

As indicated by the benchmark regression results above, DIC demonstrates a robust positive association with urban resilience. Based on the theoretical analysis above, stepwise regression is used to verify how DIC empowers urban resilience through specific mediating pathways. The mediating variables, explanatory variables, and dependent variables are substituted into Equations (2) and (3) above for regression analysis, with Table 8 presenting the results of the mediation effect tests for basic public services, technological innovation, and resource allocation. Columns (1), (3), and (5) demonstrate significantly positive coefficients for DIC’s impact on each respective mediator, confirming that DIC substantially improves basic public service provision, fosters technological innovation, and optimizes resource allocation efficiency. As shown in columns (2), (4), and (6) of Table 8, after including basic public services, technological innovation, and resource allocation, the impact coefficients of DIC on urban resilience remain significantly positive at the 1% level, suggesting that DIC enhances urban resilience by improving basic public services, technological innovation, and resource allocation capacity. Hypothesis 2 is validated. To further verify the mediating effect, the Sobel-Goodman method, drawing on the study by Ullah et al., was employed to conduct the Sobel test, Goodman 1 test, and Goodman 2 test respectively [71]. The results indicate that the outcomes of the Sobel test and Goodman tests (Goodman 1 and Goodman 2) all passed the significance test at the 1% level.

**Table 8:** Mechanism analysis results.

Variables	PS		TI		RAE	
	(1)	(2)	(3)	(4)	(5)	(6)
	*PS*	*UR*	*TI*	*UR*	*RAE*	*UR*
*DIC*	0.204***	0.219***	0.296***	0.194**	3.120***	0.223***
	(0.040)	(0.082)	(0.110)	(0.075)	(1.065)	(0.081)
*PS*		0.084***				
		(0.023)				
*TI*				0.143***		
				(0.040)		
*RAE*						0.004***
						(0.002)
Controls	Yes	Yes	Yes	Yes	Yes	Yes
_cons	0.082***	0.046***	−0.016	0.056***	2.842***	0.041***
	(0.021)	(0.014)	(0.017)	(0.013)	(0.249)	(0.015)
City fixed effect	Yes	Yes	Yes	Yes	Yes	Yes
Year fixed effect	Yes	Yes	Yes	Yes	Yes	Yes
N	3396	3396	3396	3396	3396	3396
R^2^	0.389	0.571	0.187	0.581	0.704	0.569
Sobel	0.017*** (z = 4.898)		0.042*** (z = 7.746)		0.013*** (z = 4.288)	
Goodman-1 (Aroian)	0.017*** (z = 4.873)		0.042*** (z = 7.730)		0.013*** (z = 4.259)	
Goodman-2	0.017*** (z = 4.923)		0.042*** (z = 7.763)		0.013*** (z = 4.317)	
Indirect effect	0.017*** (z = 4.898)		0.042*** (z = 7.746)		0.013*** (z = 4.288)	
Direct effect	0.219*** (z = 10.937)		0.194*** (z = 9.689)		0.223*** (z = 11.129)	
Total effect	0.236*** (z = 11.784)		0.236*** (z = 11.784)		0.236*** (z = 11.784)	
Proportion of mediation effect	7.20%		17.80%		5.51%	

Note: * p < 0.1, ** p < 0.05, *** p < 0.01, with the values in parentheses representing robust standard errors.

To further unravel the intrinsic relationships among PS, TI, and RAE, this study conducted an additional test for the chain mediating effect. As presented in Table 9, the results of the Bootstrap test reveal the composite transmission mechanism through which DIC empowers urban resilience. The indirect effect value of the path ‘DIC→PS→TI→UR’ is 0.045 (Boot SE = 0.004), with a 95% confidence interval of [0.017, 0.036] that does not include zero. This confirms the existence of the transmission chain wherein PS indirectly influence urban resilience by promoting TI. Furthermore, the path ‘DIC→PS→RAE→TI→UR’ also exhibits a significant positive effect, which unveils a complete transmission network composed of three mediating variables. Specifically, the DIC first optimizes public services, then improves resource allocation efficiency, and ultimately promotes technological innovation while enhancing urban resilience. Meanwhile, some negative chain mediating effects were also identified, such as the negative chain effect of DIC on urban resilience through PS and RAE. This may reflect the presence of a competitive inhibition effect between variables under specific conditions. When PS affect RAE through certain specific channels, or during the process where RAE influences TI, short-term efficiency loss may occur. However, in longer transmission chains, this inhibition effect is offset by positive effects, ultimately exerting a positive impact on the whole.

**Table 9:** Chain mediation bootstrap test.

Variable	Effect	Boot SE	BootLLCI	BootULCI	z	p
DIC → PS → UR	0.021	0.003	0.003	0.018	7.765	0.000
DIC → RAE → UR	0.008	0.002	0.001	0.008	5.022	0.000
DIC → TI → UR	0.142	0.016	0.050	0.109	9.043	0.000
DIC → PS → RAE → UR	−0.003	0.001	−0.003	−0.001	−5.058	0.000
DIC → PS → TI → UR	0.045	0.004	0.017	0.036	11.334	0.000
DIC → RAE → TI → UR	−0.005	0.001	−0.005	−0.000	−4.674	0.000
DIC → PS → RAE → TI → UR	0.002	0.000	0.000	0.002	5.033	0.000

Note: BootLLCI refers to the lower limit of the 95% confidence interval for Bootstrap sampling, and BootULCI refers to the upper limit of the 95% confidence interval for Bootstrap sampling.

Consequently, the three mediating effect paths proposed in this study hold valid, with partial mediating effects existing, which further verifies Hypothesis 2.

### Spatial effect analysis

The spatial spillover effects of intercity DIC on resilience are not driven by a single dimension; rather, they are jointly influenced by multiple mechanisms such as geographical proximity, economic linkages, and their complex interactions. A single matrix is insufficient to fully capture the realistic spatial interactions, whereas comparing multiple matrices can enhance the robustness and explanatory power of the findings. Therefore, this study attempts to construct three types of spatial weight matrices: adjacency matrix(*W*_*1*_), economic distance matrix(*W*_*2*_), and economic geography weight matrix(*W*_*3*_), to explore the spatial spillover effects between DIC and urban resilience. The *W*_*1*_ matrix is set based on whether cities are adjacent to each other. If cities are adjacent, the value is 1; if not, the value is 0. The *W*_*2*_ matrix is denoted by the reciprocal of the absolute difference in per – capita GDP among cities. The *W*_*3*_ matrix is expressed as the product of the reciprocal of the inter – city distance (calculated from latitude and longitude data) and the reciprocal of the absolute difference in actual GDP between regions. To evaluate spatial autocorrelation, the Moran’s I index method is used to analyze the spatial relationship between DIC and urban resilience, with results presented in Table 10. This suggests that DIC and urban resilience exhibit spatial interdependence: improvements in one city are likely to positively affect neighboring cities, highlighting the presence of spatial spillover effects in their relationship.

**Table 10:** Moran’s I index.

Year	UR			DIC		
	Adjacency matrix *W1*	Economic distance matrix *W2*	Economic-geographic weight matrix *W3*	Adjacency matrix *W1*	Economic distance matrix *W2*	Economic-geographic weight matrix *W3*
2011	0.1256***	0.3549***	0.3605***	0.1187***	0.137***	0.1353***
2012	0.1194***	0.3645***	0.3663***	0.1132***	0.1327***	0.1296***
2013	0.1398***	0.3514***	0.3586***	0.1141***	0.1686***	0.1638***
2014	0.1176***	0.3815***	0.3809***	0.0931***	0.1427***	0.1363***
2015	0.1158***	0.3548***	0.3612***	0.0802***	0.1261***	0.1163***
2016	0.1212***	0.3486***	0.3568***	0.0921***	0.1372***	0.1292***
2017	0.1173***	0.3594***	0.3664***	0.1104***	0.1318***	0.1229***
2018	0.1071***	0.3531***	0.3623***	0.0773***	0.1229***	0.1098***
2019	0.1154***	0.3363***	0.3495***	0.068**	0.1026***	0.0888***
2020	0.1322***	0.3306***	0.3417***	0.0647**	0.0908***	0.0778***
2021	0.127***	0.3217***	0.3348***	0.0712**	0.0996***	0.0935***
2022	0.1316***	0.3319***	0.3444***	0.0873***	0.1238***	0.1172***

Note: * p < 0.1, ** p < 0.05, *** p < 0.01.

LM test, Hausman test, Wald test, LR test, etc., are conducted sequentially under different spatial regression models, and finally, the individual and time two-way fixed SDM method is selected for spatial spillover effect analysis. To ensure consistency in estimation, drawing on Lee et al.‘s research [72], partial differential equations are used to decompose the total effect of DIC on urban resilience into spatial effects. Regression outcomes, presented in Table 11, reveal that across all three spatial weight matrices (*W*_*1*_, *W*_*2*_, and *W*_*3*_), DIC exerts a notably positive influence on local urban resilience. Additionally, the spatial spillover coefficient *ρ* for urban resilience is significantly positive, indicating a robust positive spatial correlation in urban resilience levels. Under each weight matrix, the direct effect values pass the 1% significance test, confirming that DIC strongly promotes local urban resilience. The spatial spillover effect of DIC on urban resilience is captured through two key components: the coefficient of the spatial lag term and the indirect effect value. Specifically, in the *W*_*1*_ matrix, the *W×DIC* coefficient is 0.122 with an indirect effect of 0.256. In the *W*_*2*_ matrix and *W*_*3*_ matrix, the *W×DIC* coefficients are 0.588 and 0.504, accompanied by indirect effects of 0.980 and 0.940, respectively. These results underscore that DIC not only bolsters local urban resilience but also creates favorable spatial spillover effects, fostering resilience in adjacent regions through both direct and indirect channels. In particular, the spillover effect is more pronounced under the weight matrix based on economic connections. In summary, DIC not only directly enhances local urban resilience but also radiates to surrounding areas through spatial spillover effects, playing an important role in promoting regional urban resilience. That is, there exists a dual pathway of “direct effect—enhancing local urban resilience” and “spatial spillover—radiating to surrounding cities’ resilience,” and this conclusion holds robust across different spatial weight matrices. Therefore, Hypothesis 3 is validated.

**Table 11:** Spatial durbin model regression results.

Variable	*UR*		
	Adjacency matrix *W1*	Economic distance matrix *W2*	Economic-geographic weight matrix *W3*
*DIC*	0.276***	0.169***	0.163***
	(0.0198)	(0.0197)	(0.0194)
*W* × *DIC*	0.122**	0.588***	0.504***
	(0.0422)	(0.0541)	(0.0573)
Direct effect	0.288***	0.202***	0.197***
	(0.0199)	(0.0197)	(0.0194)
Indirect effect	0.256***	0.980***	0.940***
	(0.0471)	(0.0689)	(0.0767)
Total effect	0.545***	1.182***	1.136***
	(0.0524)	(0.0725)	(0.0804)
Controls	Yes	Yes	Yes
City fixed effect	Yes	Yes	Yes
Year fixed effect	Yes	Yes	Yes
*ρ*	0.272***	0.357***	0.410***
	(0.0230)	(0.0290)	(0.0278)
Log-likelihood	8953.9201	9072.9968	9125.3240
R^2^	0.233	0.536	0.442

Note: * p < 0.1, ** p < 0.05, *** p < 0.01, with the values in parentheses representing robust standard errors.

## Discussion

This study systematically reveals the multi-dimensional mechanism through which DIC influences urban resilience, breaking through the traditional focus of urban resilience research on physical infrastructure and constructing a theoretical framework for enhancing urban resilience in the digital age. The findings resonate with existing literature, further extending the theoretical boundaries of how digital technologies drive sustainable urban development.

Firstly, this study finds that DIC has a significant positive impact on urban resilience, which is highly consistent with existing research on how digital infrastructure empowers sustainable urban development [66,73]. DIC facilitates the transition of cities from passive defense to proactive adaptation by enhancing the adaptive capacity and resilience of the four key subsystems—economic, social, ecological, and infrastructural—thereby expanding both the theoretical connotations and practical pathways of urban resilience.

Secondly, digital infrastructure also exerts its influence through indirect pathways, such as improving the precision of basic public services, stimulating technological innovation, and optimizing resource allocation efficiency. This aligns with the concepts of “adaptive capacity” and “transformative capacity” emphasized in urban resilience theory [32,74]. This finding extends the current understanding of the mechanisms through which digital technologies influence urban development, and it resonates with and enriches emerging paradigms in urban governance research, such as smart governance and digital twin. It demonstrates that urban digital transformation is not merely a technological upgrade, but rather a significant transformation in governance models and resource organisation methods. Furthermore, the results of the chain mediation analysis indicate that high-quality public services can lay a foundation for the release of potential in technological innovation and resource allocation. However, it is also necessary to emphasize the coordinated development of public services, technological innovation, and resource allocation efficiency, avoid resource crowding-out, and thus form a virtuous cycle of “environmental optimization, technology-driven growth, and efficiency enhancement,” thereby better facilitating the improvement of urban resilience levels.

Thirdly, an analysis of heterogeneity indicates that the facilitative role of DIC in enhancing urban resilience is more pronounced in the eastern region. This result may be attributed to the higher adoption capacity of digital technologies and industrial synergy effects in the eastern region [32]. This finding addresses the existing research gap that overlooks the dimension of “industrial compatibility” when investigating the enabling mechanisms through which DIC enhances urban resilience. Additionally, this study reveals significant differences in the effect of DIC on urban resilience based on the industrial structure of cities. The effect is more pronounced in service-dominated cities, indicating that traditional industrial cities may face higher resistance to digital transformation due to path dependency, necessitating targeted policy interventions.

Lastly, the research further validates the positive spatial diffusion impact of DIC on urban resilience. The collaboration in digital infrastructure between neighboring cities can overcome geographical distance limitations and enable the collaborative evolution of urban resilience, which aligns with the proximity effect of technological diffusion in “New Economic Geography” [75]. Furthermore, the spillover effects derived from economic linkages (W2, W3) are significantly stronger than those solely based on geographical adjacency (W1). This indicates that economic linkages not only amplify the spatial spillover effects brought by proximity but also extend the radiation scope of DIC to regions beyond physical boundaries. This is because the core value carriers of digital infrastructure are digital elements such as data, computing power, and technology. Elements like data and digital technologies incur minimal transmission costs, can be shared across regions, and their spillover effects do not rely on physical channels. The efficiency of their flow depends more on the strength of economic linkages between cities. Cities with industrial compatibility and comparable economic development levels exhibit stronger interaction and absorption capacities, thereby more effectively translating spillover effects into tangible value. Therefore, when planning the layout of digital infrastructure, it is essential to not only recognize the fundamental role of geographical proximity in facilitating coordinated development but also emphasize the key value of economic linkages in promoting and amplifying spillover effects. Priority should be given to advancing collaborative construction within urban agglomerations or metropolitan areas with close economic linkages.

### Limitations of the study and future work

Although this study reveals the mechanism by which DIC impacts urban resilience, there are still some limitations: (1) In terms of data coverage, the research primarily focuses on cities, and does not include county-level or lower administrative units, potentially overlooking the marginal effects of digital infrastructure in urban-rural integration; (2) With respect to the indicator system, and given the constraints imposed by data availability, this study did not incorporate non-traditional social indicators—such as community participation, trust, and cohesion. This omission may limit the depth and breadth of social resilience assessment, as these indicators are recognized as valuable for capturing the multidimensional nature of social resilience; (3) The study only analyzes the short-term effects from 2011 to 2022, and the long-term impact of digital infrastructure remains unclear. In the future, machine learning methods could be used to predict the dynamic evolution paths of digital infrastructure and its non-linear impact on urban resilience. To address these limitations, future research is recommended to expand in the following directions: (1) Construct multi-scale spatial econometric models that include county-level data and the synergy effects of urban agglomerations; (2) Future research will broaden data acquisition channels and employ mixed research methods, including designing targeted community questionnaires, conducting in-depth interviews with residents, and collaborating with local social work institutions to collect valid data on community participation, trust, and cohesion; (3) Introduce complexity science methods to simulate the dynamic interaction process between DIC and urban resilience systems.

## Conclusion and suggestions

This research utilizes longitudinal data spanning 2011–2022 from 283 cities across mainland China to conduct an empirical investigation into how DIC shapes urban resilience levels and its underlying mechanisms. A holistic evaluation framework for urban resilience and DIC underpins the analysis. This study yields four principal findings: First, DIC exerts a significant influence on urban resilience, with its effects more salient in economic, social, and infrastructural resilience dimensions, while ecological resilience shows no notable correlation with DIC. Second, mechanism analysis indicates that DIC can indirectly enhance urban resilience by optimizing public services, promoting technological innovation, and improving resource allocation efficiency. Third, DIC’s effect on urban resilience exhibits regional and industrial heterogeneity, with eastern cities and service-dominat cities showing stronger resilience enhancement. Fourth, digital infrastructure enables urban resilience through spatial spillover effects. While enhancing its own city’s resilience, DIC also promotes the resilience of adjacent cities.

Drawing from these research results, the study puts forward the following policy suggestions:

First, comprehensively strengthen the DIC, establishing a new cornerstone for urban resilience development. DIC serves as a pivotal driver for smart city initiatives and the “Digital China” strategy, while also acting as the bedrock for bolstering economic and social resilience. Governments at all levels should prioritize digital infrastructure advancement under the auspices of policy frameworks such as the Overall Layout Plan for Digital China Construction, Guiding Opinions on Deepening Smart City Development and Urban Digital Transformation, and Opinions on Promoting New Urban Infrastructure for Resilient City Building. Increase investments in next-generation digital infrastructures, expediting their deployment. Foster synergistic integration of transformative technologies with urban infrastructure, continuously fortifying the resilience of urban facilities, governance systems, and spatial planning to underpin urban safety.This includes accelerating the construction and deployment of high-speed networks, cloud computing platforms, and big data centers, while promoting the deep integration of cutting-edge technologies (e.g., IoT, AI) with urban infrastructure. These efforts will continuously enhance urban resilience and promote urban safety and sustainable development. Concurrently, a robust policy support system for digital infrastructure should be established, including regulations on data security and privacy protection, to create a favorable environment that enables the full potential of digital infrastructure. Through multi-dimensional policy interventions, the capacity of digital infrastructure to enhance urban resilience can be fully realized.

Second, deepen the digital transformation of public services, promote the two-way enhancement of technology-driven innovation and resource allocation efficiency, and build an intelligent modern urban management system. In public services, digital technology should be leveraged to further advance the transformation of the public service system, optimize the process and supply structure of public services, enhance the coverage and accessibility of public services, promote people’s well-being, and provide strong social support for urban resilience. In terms of technological innovation, efforts should be intensified to support the research, development (R&D), and application of digital technologies. Collaboration among enterprises, universities, and research institutions should be encouraged to achieve breakthroughs in key core technologies and to facilitate the widespread adoption of digital technologies in urban governance. In terms of resource allocation efficiency, digital technologies should be used to optimize the resource allocation process, relying on digital management platforms to improve the efficiency and transparency of resource allocation. This will provide a solid material foundation and resource guarantee for building resilient cities and maintaining the stable operation of urban systems.

Third, the enhancement of urban connectivity and cross-domain collaboration is imperative to establish a novel paradigm of regional coordinated development. The layout of DIC must shift from the physical thinking of “spreading nodes and connecting networks” to the systematic thinking of “weaving networks and empowering functions”. It is essential to construct a digital community linked by economic connections and industrial collaboration, proactively shape and strengthen the economic functional connections among cities, and maximize the potential of digital infrastructure in enhancing the resilience of cities at both the national and regional levels. Eastern regions and service-oriented cities, which have demonstrated substantial leadership advantages in DIC, should continue to deepen the integration of digital technologies with urban governance. They should establish demonstration benchmarks for smart cities, actively explore innovative application scenarios, and further enhance the refinement and intelligence of governance, thereby providing replicable advanced experiences and models for other regions. Central and western regions, along with industry-dominated cities, should strengthen policy guidance and give priority in resource allocation. Through targeted measures such as special subsidies for digital infrastructure and tax incentives, they should prioritize the development of key sectors, including 5G base stations, the industrial Internet, and data centers. Meanwhile, they should promote the in-depth application of digital technologies in economic operations, social governance, and the transformation and upgrading of traditional industries, so as to effectively address the shortcomings in building urban resilience in these regions. Furthermore, cross-regional coordination mechanisms should be established. By leveraging the network advantages of digital infrastructure, cities can enhance inter-city policy communication, information sharing, and institutional co-construction to improve regional synergy. Through technology transfer, talent cultivation, and project cooperation from eastern to central and western regions, high-quality digital resources can be promoted in less developed areas, thereby gradually narrowing the regional development gap and achieving an overall improvement in urban resilience.

## Supporting information

S1 File The supplementary supporting information of this study has been compiled in the Excel file of the supplementary materials. (XLSX)
